# Supplementary material for: Bifunctional Peptidomimetic G Protein-Biased Mu-Opioid Receptor Agonist and Neuropeptide FF Receptor Antagonist KGFF09 Shows Efficacy in Visceral Pain without Rewarding Effects after Subcutaneous Administration in Mice
Source: Molecules. 2022 Dec 11;27(24):8785. doi: 10.3390/molecules27248785 (PMC9780937; doi:10.3390/molecules27248785)
Supplement: Supplementary file 1 [file molecules-27-08785-s001.zip › molecules-2026276-supplementary.pdf]

Supporting Material

Bifunctional peptidomimetic G protein-biased mu-opioid receptor agonist and neuropeptide FF receptor antagonist KGFF09 shows efficacy in visceral pain without rewarding effects after subcutaneous administration in mice

Maria Dumitrascuta[1], Charlotte Martin[2], Steven Ballet[2], Mariana Spetea[1,*]

[1]Department of Pharmaceutical Chemistry, Institute of Pharmacy and Center for Molecular Biosciences Innsbruck (CMBI), University of Innsbruck, Innrain 80-82, 6020 Innsbruck, Austria; maria.dumitrascuta@uibk.ac.at (M.D.)
[2]Departments of Chemistry and Bioengineering Sciences, Vrije Universiteit Brussel, Pleinlaan 2, B-1050 Brussels, Belgium; charlotte.martin@vub.be (C.M.) and steven.ballet@vub.be (S.B.)

*Correspondence: mariana.spetea@uibk.ac.at (M.S.)

Table S1. In vitro profile of **KGFF09** to the human opioid and NPFF receptors[a]

**Receptor**	**Binding affinity**[b]	**Functional activity G protein activation**[c]			**Functional activity β-arrestin2 recruitment**[d]	
	**K_i (nM)**	**EC_{50} (nM)**	**E_{max} (%)**	**pA2**	**EC_{50} (nM)**	**E_{max} (%)**
MOR	2.43	18.2	85		1264	57
DOR	186	0.79	90			
KOR	3.2	>10000	15			
NOP receptor	209	>10000	7			
NPFF1R	83		7.25			
NPFF2R	3.2			7.77		

[a]Data from ref. [1]. [b]Determined in radioligand competitive binding assays. [c]Determined in the cAMP accumulation assay (opioid receptors) or [^{35}S]GTPγS binding assay (NPFF receptors). [d]Determined in the BRET β-arrestin-2 recruitment assay.

Table S2. In vivo profile of **KGFF09** in mice after s.c. administration

**Behavioral response**[a]	**Effect**
**Antinociception**	
Acute pain model	
warm-water tail withdrawal test[a]	yes
radiant tail-flick test[a]	yes
Chronic inflammatory pain model	
CFA-induced hyperalgesia[a]	yes
Visceral pain model	
Acetic acid-induced writing assay[b]	yes
**Unwanted side effects**[c]	
Acute administration	
respiratory depression[a]	no
constipation[a]	yes
sedation/motor impairment[a]	yes
Chronic administration	
opioid-induced hyperalgesia[a]	no
antinociceptive tolerance[a]	no
withdrawal syndrome[a]	no
reward[b]	no
hyperlocomotion[b]	no

[a]Data from ref. [1]. Determined in this study. [c]Determined at effective antinociceptive doses.

Table S3. Comparison of potencies and efficacies to the human MOR of **KGFF09** in functional in vitro assays for G protein activation and β-arrestin2 recruitment

**Ligand**	**G protein activation**				**β-arrestin2 recruitment**			
	**cAMP accumulation**[a]		**[^{35}S]GTPγS binding**[b]		**BRET β-arrestin2 recruitment**[a]		**PathHunter β-arrestin2 recruitment**[c]	
	**EC_{50} (nM)**	**E_{max} (%)**	**EC_{50} (nM)**	**E_{max} (%))**	**EC_{50} (nM)**	**E_{max} (%)**	**EC_{50} (nM)**	**E_{max} (%)**
KGFF09	18.2	85	8.28	92	56.4	42	1264	57
DAMGO	80.4	100	35.0	100	240	100	367	100

[a]Data from ref. [1]. [b]Determined in the [^{35}S]GTPγS binding assay with membranes from CHO cells stably expressing the human MOR. [c]Determined in the PathHunter β-arrestin2 recruitment assay with U2OS cells co-expressing the human MOR and the enzyme acceptor tagged β-arrestin2 fusion protein. E_{max} (%) values represent percentage relative to the maximal effect of DAMGO (as 100%).

Reference

1. Drieu La Rochelle, A.; Guillemyn, K.; Dumitrascuta, M.; Martin, C.; Utard, V.; Quillet, R.; Schneider, S.; Daubeuf, F.; Willemse, T.; Mampuys, P.; Maes, B.U.W.; Frossard, N.; Bihel, F.; Spetea, M.; Simonin, F.; Ballet, S. A bifunctional-biased mu-opioid agonist-neuropeptide FF receptor antagonist as analgesic with improved acute and chronic side effects. *Pain* **2018**, 159, 1705-1718.
